# Pharmacogenomics and non-genetic factors affecting drug response in autism spectrum disorder in Thai and other populations: current evidence and future implications

**DOI:** 10.3389/fphar.2023.1285967

**Published:** 2024-02-05

**Authors:** Mohitosh Biswas, Natchaya Vanwong, Chonlaphat Sukasem

**Affiliations:** ^1^ Department of Pharmacy, University of Rajshahi, Rajshahi, Bangladesh; ^2^ Division of Pharmacogenomics and Personalized Medicine, Department of Pathology, Faculty of Medicine Ramathibodi Hospital, Mahidol University, Bangkok, Thailand; ^3^ Laboratory for Pharmacogenomics, Ramathibodi Hospital, Somdech Phra Debaratana Medical Center SDMC, Bangkok, Thailand; ^4^ Department of Clinical Chemistry, Faculty of Allied Health Sciences, Chulalongkorn University, Bangkok, Thailand; ^5^ Cardiovascular Precision Medicine Research Group, Special Task Force of Activating Research (STAR), Chulalongkorn University, Bangkok, Thailand; ^6^ Pharmacogenomics and Precision Medicine Clinic, Bumrungrad Genomic Medicine Institute (BGMI), Bumrungrad International Hospital, Bangkok, Thailand; ^7^ Faculty of Pharmaceutical Sciences, Burapha University, Mueang, Thailand; ^8^ Department of Pharmacology and Therapeutics, MRC Centre for Drug Safety Science, Institute of Systems, Molecular, and Integrative Biology, University of Liverpool, Liverpool, United Kingdom

**Keywords:** autism spectrum disorder, risperidone, aripiprazole, pharmacokinetic/pharmacodynamic, genetic polymorphisms, pharmacogenomics, precision medicine

## Abstract

Autism spectrum disorder (ASD) may affect family and social life profoundly. Although there is no selective pharmacotherapy for ASD, the Food and Drug Administration (FDA) has recommended risperidone/aripiprazole to treat the associated symptoms of ASD, such as agitation/irritability. Strong associations of some pharmacokinetic/pharmacodynamic gene variants, e.g., *CYP2D6* and *DRD2*, with risperidone-induced hyperprolactinemia have been found in children with ASD, but such strong genetic associations have not been found directly for aripiprazole in ASD. In addition to pharmacogenomic (PGx) factors, drug–drug interactions (DDIs) and possibly cumulative effects of DDIs and PGx may affect the safety or effectiveness of risperidone/aripiprazole, which should be assessed in future clinical studies in children with ASD. Reimbursement, knowledge, and education of healthcare professionals are the key obstacles preventing the successful implementation of ASD pharmacogenomics into routine clinical practice. The preparation of national and international PGx-based dosing guidelines for risperidone/aripiprazole based on robust evidence may advance precision medicine for ASD.

## 1 Introduction

Autism spectrum disorder (ASD) can be categorized as “syndromic ASD,” which is associated with morphological signs or symptoms, e.g., restricted, repetitive, and stereotyped patterns of behavior, etc., or as “non-syndromic ASD,” alternatively termed idiopathic ASD, which has no associated signs or symptoms (Genovese and Butler, 2020; Aishworiya et al., 2022). The core clinical features of ASD are difficulties in social communication, restricted or fixated interests, and language delays or speech difficulties (Sauer et al., 2021). ASD may affect family and social life profoundly; therefore, it is important to screen all infants and toddlers to identify early signs of ASD at 9 months, 18 months, and again at 24 or 30 months of age, as recommended by the American Academy of Pediatrics (AAP). Well-established and validated rating or assessment scales should be applied for the diagnosis of ASD, such as the scales of the Autism Diagnostic Interview-Revised (ADI-R) and the Autism Diagnostic Observation Schedule, Second Edition (ADOS-2). Along with the consideration of the history and clinical presentation of the child, these scales should be applied by trained specialists for the evaluation of ASD (Genovese and Butler, 2020; Aishworiya et al., 2022; Biswas et al., 2022b).

Another well-known 20-point assessment scale is the Modified Checklist for Autism in Toddlers-Revised (M-CHAT-R), developed by the American Association for Child and Adolescent Psychiatry (AACAP). The AACAP recommends checking the risk of ASD through surveillance using this assessment scale for children at the age of 18 and 24 months or when such assessment becomes necessary (Subramanyam et al., 2019). The child is predicted to be at low risk, medium risk, or high risk of ASD if the assessment total score is 0–2, 3–7, or 8–20, respectively. Early screening of symptomatic biomarkers, including developmental, behavioral, cognitive, and body movement/motor developmental-related markers, as described by Subramanyam et al. (2019), may help detect significant ASD symptoms. A thorough diagnostic evaluation is warranted if early detection of ASD symptoms is confirmed (Subramanyam et al., 2019). Recently, Magellan Health adopted clinical practice guidelines for the assessment and treatment of children with ASD that extensively discuss the epidemiology, diagnosis, comorbidity, assessment, pharmacotherapy, and educational and behavioral interventions (Ghani et al., 2020).

Genetic factors governing the predisposition of ASD are under investigation and continue to be firmly established. Some copy number variations (CNVs) may have been associated with the increased risk of developing ASD (Bernier et al., 2016; Zarrei et al., 2019; Bauleo et al., 2021; Costa et al., 2022). A recent whole-exome sequencing study identified one *de novo* causative variant (c.2951G>A) in the *FGD6* gene (OMIM ID: 613520) in Thai ASD patients (Thongnak et al., 2018).

As reviewed by the WHO in 2012, the estimated prevalence of ASD was ∼1% globally, although the prevalence rate has been slightly higher (∼1·5%–2%) in recent years, as revealed by Lord et al. (2018), DeVane et al. (2019), Turner (2020), and Biswas et al. (2022b). In most developed countries, the distribution of ASD patients shows similar patterns; however, it is comparatively less prevalent in low- and lower-middle-income countries (Biswas et al., 2022b). The increase in the prevalence rate in Western countries over the past several decades has been partly due to changes in diagnostic methods and the inclusion criteria of ASD. Approximately 1 out of 59 children was diagnosed with ASD in the United States of America, and 205,200 children in Australia were diagnosed with ASD in 2018, which represents a ∼25% increase in the prevalence rate than that reported in 2015 (Lord et al., 2018; Hyman et al., 2020; Turner, 2020).

However, studies collecting epidemiological data relevant to ASD from low- and lower-middle-income countries are very limited (Hyman et al., 2020). The prevalence rate of ASD in many of these countries is still unknown (Elsabbagh et al., 2012).

There may be some comorbidities or clinical features associated with ASD, including electroencephalogram (EEG) abnormalities with or without epilepsy, intellectual disability (ID), and abnormal findings on magnetic resonance imaging (MRI). Approximately 10% of children with ASD have microcephaly, 28% have attention-deficit/hyperactivity disorder (ADHD), 20% have anxiety disorders, 13% have insomnia disorders, 11% have depressive disorders, 9% have obsessive–compulsive disorder, 5% have bipolar disorders, and 4% have schizophrenia spectrum disorders, as described in some studies. Head enlargement is also common in children with ASD, along with higher brain volumes, especially in the frontal lobes (Genovese and Butler, 2020; Turner, 2020).

Pharmacogenomics (PGx) aims to optimize the overall safety and effectiveness of many clinically recommended conventional medications, considering the genetic variants of drug-metabolizing enzymes, such as cytochrome P450 (CYP) enzymes, or transporter biomolecules affecting the pharmacokinetic or pharmacodynamic properties of the drugs, as evidenced in various studies (Roden et al., 2006; Somogy, 2008; Whirl-Carrillo et al., 2012; Ahmed et al., 2016; Collins et al., 2016; Zhou et al., 2017; Chidambaran and Sadhasivam, 2018; Biswas et al., 2023). It is now well recognized that a ‘one-size-fits-all’ approach will not be effective for many clinically important medications for certain groups of patients carrying either *CYP* or transporter genetic variants. Instead, a more personalized treatment approach, called precision medicine, achieved through considerations of PGx, is now clinically feasible and operational in many parts of the world (Collins and Varmus, 2015; Relling, 2015; Relling and Evans, 2015; Weinshilboum and Wang, 2017; Blagec et al., 2018; Biswas et al., 2021a; Biswas, 2021; Gong et al., 2021; Sukasem et al., 2023). This narrative review aims to address the therapeutic guidelines, pharmacokinetic/pharmacodynamic properties of ASD medications, current evidence of PGx for ASD medications, and non-genetic factors affecting the safety or effectiveness of ASD medications.

## 2 Therapeutic guidelines for ASD

ASD, with its complex biological traits, can be difficult to diagnose, especially at the initial stage. Therefore, clinical treatments aimed at compensating for the symptoms associated with ASD are not straightforward. Currently, there are no robust guidelines to follow for ameliorating the symptoms of ASD. However, psychostimulants, atypical antipsychotics, antidepressants, and alpha-2 adrenergic receptor agonists are commonly used to treat core clinical symptoms or manage the symptoms of comorbid conditions in children and adolescents with ASD, as reported by Sharma et al. (2018). The United States Food and Drug Administration (FDA) has approved two drugs, risperidone and aripiprazole, not for the treatment of ASD directly but for alleviating the irritability or agitation symptoms associated with ASD in children and adolescents aged 5–16 years. Risperidone was approved in 2006 by the FDA at a typical dose of 1–3 mg/day. In 2009, aripiprazole was ratified by the FDA at a typical dose of up to 15 mg/day (Riesgo, R., Gottfried, C., & Becker, 2013; Lord et al., 2018; DeVane et al., 2019; Hongkaew et al., 2021b; Biswas et al., 2022b).

## 3 Clinical problems in ASD treatment

There is no selective therapy for treating the core symptoms of ASD; however, co-occurring health problems commonly reported in ASD, such as attention-deficit/hyperactivity disorder, irritability, agitation, epilepsy, sleep disorders, anxiety, and depression, are usually treated with supportive treatments (Howes et al., 2018; Biswas et al., 2022b). For example, risperidone/aripiprazole is commonly used as a first-line therapy to treat irritability or agitation associated with ASD. However, several adverse effects, e.g., weight gain, increased prolactin level in the blood (hyperprolactinemia), hyperuricemia, leptin disturbance, insulin resistance, and extrapyramidal effects, are commonly reported with the use of risperidone/aripiprazole in children with ASD (Germann et al., 2012; Vanwong et al., 2016c; 2020; Shafiq and Pringsheim, 2018; Aishworiya et al., 2022; Biswas et al., 2022b). Patients might also be at risk of the therapeutic ineffectiveness of the drug. For example, risperidone can be metabolized by the CYP2D6 enzyme, and some phenotypes are potentially being considered CYP2D6 ultra-rapid metabolizers due to the rapid clearance of this drug from the body, as described by Biswas et al. (2022b).

## 4 Psychopharmacological treatments for ASD

Although the core treatment of ASD is largely dependent on effective behavioral interventions, several potential supportive treatments targeting the underlying neurological disorders of ASD have been the mainstay of ASD management over the last few years (Aishworiya et al., 2022). It has been reported that approximately two-thirds of adolescent ASD patients have been treated with psychotropic medications, especially those diagnosed with neuropsychological problems. Approximately 70% of ASD patients have been found to have several other problems, such as ADHD, irritability, aggression, and mood and anxiety issues, warranting the use of psychotropic medications in these patients (Simonoff et al., 2008; Levy et al., 2010; Feroe et al., 2021; Aishworiya et al., 2022). The following medications are frequently prescribed to ASD children (Aishworiya et al., 2022).

**Risperidone:** This drug was approved by the FDA in 2006 for children with ASD. It can be prescribed for children older than 5 years of age to reduce the irritability associated with ASD (Riesgo, R., Gottfried, C., & Becker, 2013; Lord et al., 2018; DeVane et al., 2019; Hongkaew et al., 2021b; Biswas et al., 2022b).

**Aripiprazole:** The FDA approved aripiprazole in 2009 for ASD children who were 6–17 years of age for reducing irritability (Owen et al., 2009; Riesgo, R., Gottfried, C., & Becker, 2013; Lord et al., 2018; DeVane et al., 2019; Hongkaew et al., 2021b; Aishworiya et al., 2022; Biswas et al., 2022b).

**Serotonin reuptake inhibitors, anti-anxiety medications, or stimulants:** Although the FDA has not recommended the use of selective serotonin reuptake inhibitors (SSRIs, e.g., sertraline, citalopram, fluoxetine, and venlafaxine), tricyclic antidepressants (TCAs, e.g., amitriptyline and nortriptyline), or stimulants (e.g., amphetamine and methylphenidate) for ASD, some studies have suggested using these medications for certain clinical benefits (Aishworiya et al., 2022). For example, SSRIs may stimulate neurogenesis and produce a neuroprotection effect in ASD children; therefore, some clinicians prefer to use these medications, especially to treat anxiety, mood issues, and irritability associated with ASD (Aishworiya et al., 2022).

**Anticonvulsants**: Almost one-third of people with ASD have seizures or seizure disorders (Hirota et al., 2014; Besag and Vasey, 2021). Antiepileptic drugs, e.g., carbamazepine and lamotrigine, are commonly prescribed for ASD alongside seizures or seizure disorders. Clinical effectiveness remains controversial (Hirota et al., 2014).

## 5 Risperidone

### 5.1 Pharmacokinetics of risperidone

**FIGURE 1:**
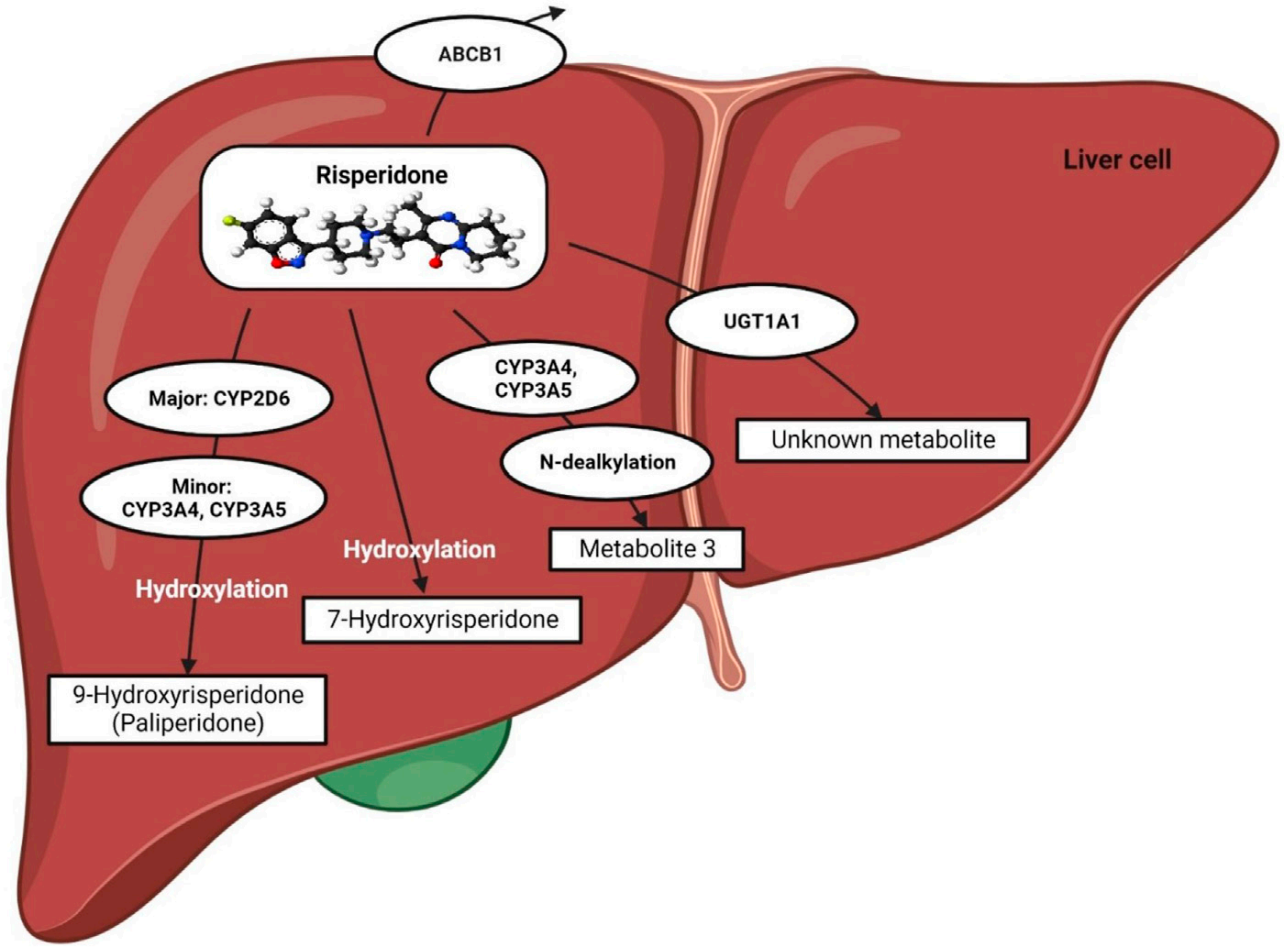
Metabolic pathway of risperidone. This figure is adapted from Biswas et al., (Biswas et al., 2022b).

The metabolic pathway of risperidone (a pro-drug) was extensively discussed in our previous review (Biswas et al., 2022b). In short, it is preferentially metabolized by CYP2D6 to a greater extent, whereas CYP3A4/5 enzymes might play a minor role in producing 9-hydroxyrisperidone, known as paliperidone, which is a pharmacologically active moiety (Fang et al., 1999; Berecz et al., 2004; Corena-McLeod, 2015; Puangpetch et al., 2016; Biswas et al., 2022b) (Figure 1).

Risperidone may also act as a substrate and an inhibitor of P-glycoprotein (P-gp), as reported in recent *in vitro* studies (Puangpetch et al., 2016; Soria-Chacartegui et al., 2021). Furthermore, UGT1A1 may also be potentially involved in the metabolic pathway of risperidone since an association between *UGT1A1* genetic polymorphisms and risperidone-induced hyperprolactinemia has been established in a clinical study in Thailand (Hongkaew et al., 2018).

### 5.2 Pharmacodynamics of risperidone

**FIGURE 2:**
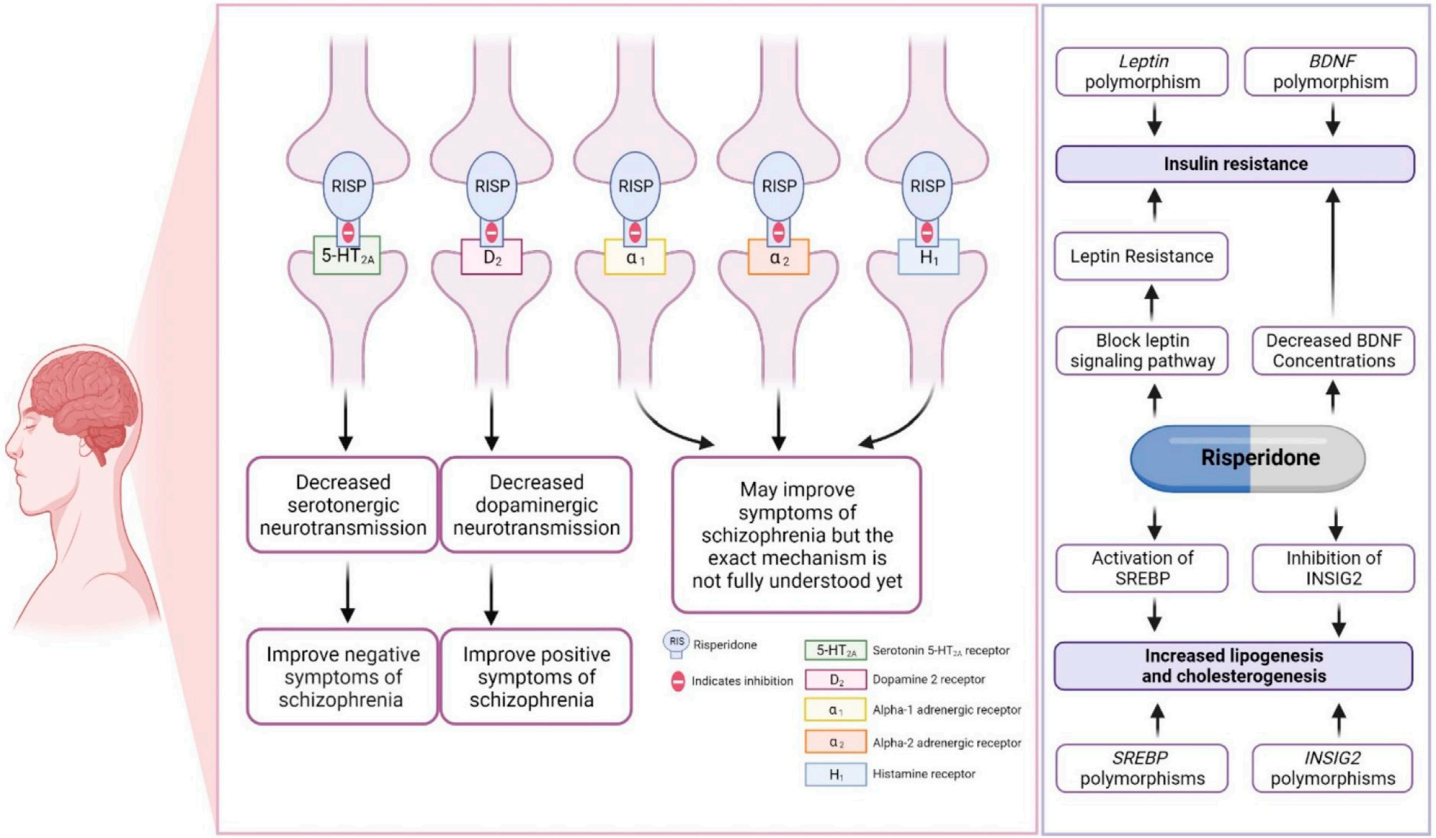
Pharmacodynamics of risperidone. This figure is adapted from Biswas et al., (Biswas et al., 2022b).

Risperidone primarily antagonizes the serotonergic (5-HT_2A_) and dopaminergic (D2) receptors in the brain, although the exact mechanism is not yet fully understood. Risperidone binds ∼10–20-fold more preferentially to 5-HT_2A_ receptors than to D2 receptors, and it is considered a potent 5-HT_2A_ receptor antagonist (Leysen et al., 1988; Fenton and Scott, 2005; Canitano and Scandurra, 2008; Kemp et al., 2009; Germann et al., 2012; Corena-McLeod, 2015; Puangpetch et al., 2016; Chopko and Lindsley, 2018; Biswas et al., 2022b). The mechanism by which risperidone reduces associated symptoms of ASD was discussed in detail in our previous review (Biswas et al., 2022b), as shown in Figure 2. Other pharmacodynamic targets, such as brain-derived neurotrophic factor (BDNF) and leptin (LEP), may also be involved in risperidone-induced insulin resistance (Figure 2).

### 5.3 Association of *BDNF* and leptin genetic variants with insulin resistance

The *BDNF* gene (OMIM ID: 113505) encoding brain-derived neurotrophic factor (BDNF) and the *LEP* gene (OMIM ID: 164160) encoding leptin (LEP) were found to have a significant association with insulin resistance in ASD patients taking risperidone, suggesting a genetic biomarker for predicting insulin resistance in ASD patients. This association must be replicated in future studies (Sukasem et al., 2018). BDNF has been reported to influence glucose–insulin homeostasis (Tsuchida et al., 2001). Previous studies have reported that decreased BDNF concentrations are found in type 2 diabetes patients (Nakagawa et al., 2000; Krabbe et al., 2007). Interestingly, administration of risperidone has been associated with decreased BDNF levels in the rat brain (Angelucci et al., 2000). The reduction in brain BDNF after being treated with risperidone, along with *BDNF* gene polymorphisms, might be a part of the mechanism causing risperidone-induced type 2 diabetes in people with autism spectrum disorder.

The LEP hormone is a regulator of glucose homeostasis and insulin resistance (Park and Ahima, 2015). Risperidone could reduce both leptin-induced signal transducer and activator of transcription 3 (STAT3) phosphorylation and insulin-mediated protein kinase B activation, which could result in LEP and insulin resistance (Piao et al., 2014). Genetic polymorphisms in *LEP* may affect the safety of risperidone. Although not widely assessed clinically, one study established an association between a SNP of *LEP* (rs7799039) and an increased risk of weight gain in ASD patients taking risperidone (Dos Santos-Júnior et al., 2016; Biswas et al., 2022b). Since the associations have not yet been assessed and replicated in other studies, further studies are needed to confirm the associations of *BDNF* or *LEP* genetic variants with insulin resistance/weight gain in ASD patients treated with risperidone.

### 5.4 Association of *INSIG2* and *SREBF2* genetic variants with dyslipidemia

*Sterol regulatory element binding transcription factor 2 (SREBF2)* gene (OMIM ID: 600481) and *insulin-induced gene2* (*INSIG2*) (OMIM ID: 608660) polymorphisms were found to be associated with dyslipidemia in patients treated with risperidone (Vanwong et al., 2021). The *SREBF2* and *INSIG2* genes are involved in the regulation of lipid biosynthesis (DeBose-Boyd and Ye, 2018). Risperidone stimulates both lipogenesis and cholesterogenesis through INSIG2 inhibition and the activation of SREBP2 expression (Cai et al., 2015). *SREBF2* and *INSIG2* might be candidate genes for dyslipidemia in people with autism spectrum disorder treated with risperidone. However, it is necessary to replicate such associations in future studies.

### 5.5 Clinical outcomes: response and adverse drug reactions of risperidone

The clinical responses and adverse effects of risperidone largely depend on the functional activity of the CYP2D6 enzyme, since risperidone is mainly metabolized by CYP2D6. Some of the less-serious adverse effects of risperidone that are commonly reported are insomnia, anxiety, decreased libido, sedation, dystonia, blurred vision, tachycardia, hypotension/hypertension, and musculoskeletal pain (Soria-Chacartegui et al., 2021; Biswas et al., 2022b). However, the more serious adverse effects of risperidone are weight gain, insulin resistance, hyperprolactinemia, and extrapyramidal effects. These adverse effects may be governed by genetic variants modifying the pharmacokinetic or pharmacodynamic properties of risperidone (Biswas et al., 2022b).

## 6 Aripiprazole

Aripiprazole, like risperidone, is an atypical antipsychotic mainly used to treat schizophrenia and bipolar disorder; however, it can be used for the management of major depressive disorder and irritability associated with ASD (Dean and Kane, 2012).

### 6.1 Pharmacokinetics of aripiprazole

**FIGURE 3:**
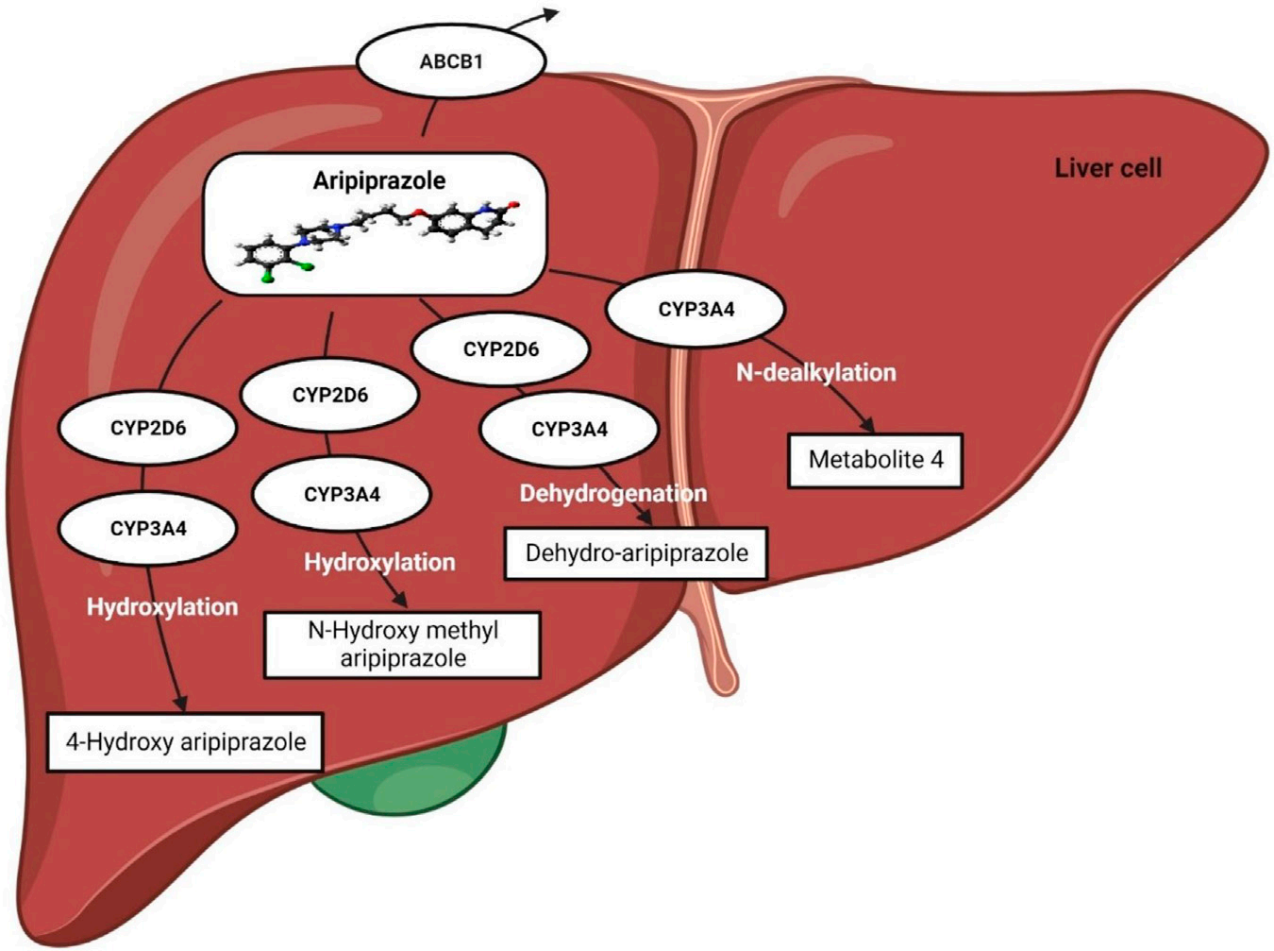
Metabolic pathway of aripiprazole.

Aripiprazole is extensively metabolized in the liver, predominantly by the CYP2D6 and CYP3A4 metabolic enzymes, and converted to dehydroaripiprazole (major metabolite) (Figure 3). The pharmacological activity of aripiprazole is primarily mediated through the parent drug; however, dehydroaripiprazole plays a very minor role in its activity. Aripiprazole takes ∼75 h to be eliminated from the body in a normal individual; however, for individuals with poor CYP2D6 activity, i.e., poor metabolizers, it takes ∼146 h to be eliminated (Dean and Kane, 2012). It has been reported that in poor metabolizers, the mean aripiprazole exposure may be increased 1.5-fold compared to normal metabolizers (Dean and Kane, 2012; Jukic et al., 2019).

### 6.2 Pharmacodynamics of aripiprazole

Unlike risperidone, aripiprazole acts as a partial agonist of the dopamine receptor (D2) and has a high affinity for binding like dopamine. However, due to the low intrinsic activity of aripiprazole, it causes very low activation of the D2 receptor compared with dopamine, which favors its use against psychiatric problems. Aripiprazole may reduce the activity of dopamine neurons profoundly in the brain’s mesolimbic system due to its high affinity for the D2 receptor and its partial agonist activity. Since overactivity of dopamine causes psychosis and other psychiatric problems, the reduction of dopamine in this region is clinically beneficial in these patients (Potkin et al., 2003; Swainston Harrison and Perry, 2004; Dean and Kane, 2012). In addition, aripiprazole exhibits a strong binding affinity for both 5-HT1A and 5-HT2A receptors. When it binds to the 5-HT1A receptor, aripiprazole acts as a partial agonist, whereas it functions as an antagonist at the 5-HT2A receptor. This mechanism of action could potentially explain the anxiolytic and anti-depressive effects of aripiprazole, as well as its ability to improve cognitive functioning and negative symptoms (Hoyer et al., 2002; Gründer et al., 2006; Dean and Kane, 2012; Muneer, 2016), Figure 4.

**FIGURE 4:**
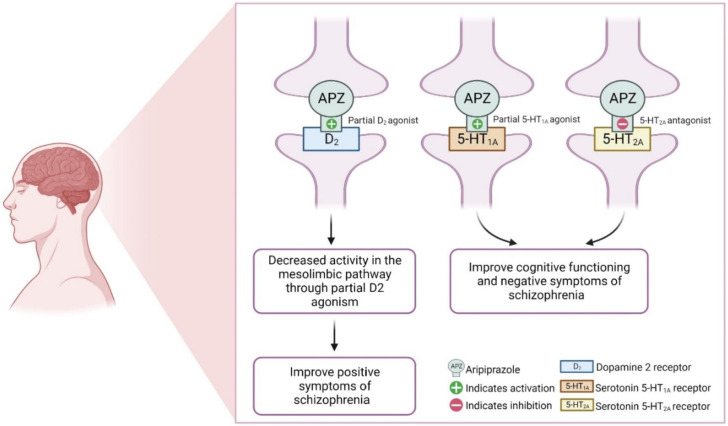
Pharmacodynamics of aripiprazole.

### 6.3 Clinical outcomes: response and adverse drug reactions of aripiprazole

Due to the 5-HT_2A_ antagonistic and D2 agonist activity of aripiprazole, it is primarily indicated in schizophrenia and is also used to treat irritability associated with ASD. Some of the common adverse effects of aripiprazole are suicidal tendencies, neuroleptic malignant syndrome, hyperglycemia, orthostatic hypotension, leukopenia, neutropenia, agranulocytosis, seizures/convulsions, sedation, and extrapyramidal disorders. (Dean and Kane, 2012).

## 7 Pharmacogenomics in ASD

### 7.1 Pharmacogenomics of risperidone

#### 7.1.1 Association of *CYP2D6* genetic variants with risperidone-induced hyperprolactinemia

Since CYP2D6 is the major CYP enzyme involved in risperidone metabolism, the safety or efficacy of risperidone may be affected by *CYP2D6* (OMIM ID: 124030) genetic variants encoding the CYP2D6 enzyme (Puangpetch et al., 2016; de Leon, 2020; Lu et al., 2021; Soria-Chacartegui et al., 2021; Biswas et al., 2022b). Patients who are considered to be CYP2D6 poor metabolizers (PMs) or CYP2D6 intermediate metabolizers (IMs) carrying defective *CYP2D*6 alleles might develop higher plasma concentrations of the risperidone/9-hydroxyrisperidone ratio compared to patients considered normal CYP2D6 metabolizers (NMs) or ultra-rapid metabolizers (UMs) (Puangpetch et al., 2016; Soria-Chacartegui et al., 2021). As a consequence, ASD children who are CYP2D6 PMs or IMs might be at higher risk of developing hyperprolactinemia when treated with risperidone (Puangpetch et al., 2016; de Leon, 2020; Soria-Chacartegui et al., 2021; Biswas et al., 2022b). In contrast, patients considered CYP2D6 UMs might be at risk of therapeutic ineffectiveness/failure of risperidone therapy due to a possible reduction of risperidone/9-hydroxyrisperidone plasma concentrations in these phenotypes (Soria-Chacartegui et al., 2021; Biswas et al., 2022b).

#### 7.1.2 Association of *UGT1A1* genetic variants with risperidone-induced hyperprolactinemia

Although the exact metabolic role of UGT1A1 in risperidone metabolism has not yet been elucidated, a recent study found a significant association of hyperprolactinemia with *UGT1A1* (OMIM ID: 191740) genetic polymorphisms in 84 Thai patients with ASD (Hongkaew et al., 2018). Therefore, it is suggested to replicate these findings in other clinical studies of ethnically diverse ASD patients.

#### 7.1.3 Association of *DRD2* genetic variants with risperidone-induced hyperprolactinemia

Since dopamine–D_2_-receptor (DRD2) is a pharmacodynamic target of risperidone, the *DRD2* (OMIM ID: 126450) gene encoding this receptor may be associated with risperidone-induced safety or efficacy issues for the patients taking this drug. Lately, a significant association between *DRD2* genetic polymorphisms and hyperprolactinemia has been established in children with ASD (Sukasem et al., 2016; Hongkaew et al., 2021a; Soria-Chacartegui et al., 2021).

#### 7.1.4 Association of *LEP* genetic variants with risperidone-induced weight gain

Genetic polymorphisms in *LEP* may affect the safety of risperidone. It has been found that ASD patients taking risperidone and harboring the rs7799039 SNP of *LEP* (GG genotype) have an increased risk of weight gain compared to AA/AG genotypes (Dos Santos-Júnior et al., 2016; Biswas et al., 2022b).

Some other genetic variants, e.g., *ABCB1*, *HTR2C* (OMIM ID: 312861), *CYP3A4/5* (OMIM ID: 124010/605325), and *CNR1* (OMIM ID: 114610), may also affect the safety or effectiveness of risperidone, as reviewed by our group (Biswas et al., 2022b). These pharmacogenomic associations should be replicated in ASD cohorts in future investigations.

#### 7.1.5 Pharmacogenomics of aripiprazole

Since aripiprazole is preferentially metabolized by CYP2D6, the safety or effectiveness of this drug might be affected by *CYP2D6* genetic variability. Recent studies have found an association between *CYP2D6* genetic polymorphisms and hyperprolactinemia, especially in female pediatric populations who are poor *CYP2D6* metabolizers (Grădinaru et al., 2019; Koller et al., 2020). Hyperprolactinemia may significantly affect the growth and development of pediatric populations (Grădinaru et al., 2019), and therefore, these patients need additional monitoring, especially when diagnosed with ASD. Recent case reports found an association between *CYP2D6* activity and atrial fibrillation or abnormal heart electrophysiology (D’Urso et al., 2018; Mazer-Amirshahi et al., 2019), suggesting that *CYP2D6* genetic variants affecting this enzyme activity should be considered in future studies.

#### 7.1.6 Pharmacogenomics of carbamazepine

Since epilepsy appears to be prevalent in ASD patients, antiepileptic drugs, e.g., carbamazepine, might be commonly prescribed to children with ASD. The pharmacogenomic response of carbamazepine in ASD patients has not been assessed yet; however, an association of *HLA-B* (OMIM ID: 142830) pharmacogenomics with carbamazepine-induced SJS/TEN has already been well established (Kloypan et al., 2021; Biswas et al., 2022a) and needs further consideration in ASD patients.

#### 7.1.7 Pharmacogenomics of SSRIs and methylphenidate

Although there is strong evidence for the pharmacogenomic effects of SSRIs, e.g., sertraline, citalopram, and escitalopram, due to *CYP2C19* (OMIM ID: 124020) and *CYP2D6* genetic variability (Hicks et al., 2015), these effects have not been quantified in children with ASD. Future studies should consider these genetic variants in ASD. A recent meta-analysis found statistically significant associations between *ADRA2A* (OMIM ID: 104210), *COMT* (OMIM ID: 116790), and *SLC6A2* (OMIM ID: 163970) genetic variants and the effectiveness of methylphenidate; however, these associations were not pooled from ASD patients (Myer et al., 2018). These genetic variants should be assessed in ASD patients taking methylphenidate.

Clinical annotations of drugs potentially used in ASD with a PharmGKB evidence level are shown in Table 1.

**TABLE 1:** Clinical annotations and gene polymorphisms of drugs potentially used in ASD.

Level	Variant	Gene	Drug	Phenotype category	Phenotype
Level 1A	CYP2D6*1, CYP2D6*1xN, CYP2D6*3, CYP2D6*4, CYP2D6*5, CYP2D6*6, CYP2D6*10, CYP2D6*14	*CYP2D6*	Risperidone	Metabolism/PK	Psychotic disorders and schizophrenia
Level 1A	CYP2D6*1, CYP2D6*4, CYP2D6*5, CYP2D6*6, CYP2D6*10, CYP2D6*41	*CYP2D6*	Aripiprazole	Metabolism/PK	Psychotic disorders, schizoaffective disorder, and schizophrenia
Level 1A	CYP2D6*1 , CYP2D6*1xN , CYP2D6*2 , CYP2D6*2xN , CYP2D6*3 , CYP2D6*4 , CYP2D6*5 , CYP2D6*, CYP2D6*10 , CYP2D6*14 , CYP2D6*41	*CYP2D6*	Paroxetine	Metabolism/PK	-
Level 1A	CYP2D6*1, CYP2D6*4, CYP2D6*5, CYP2D6*6, CYP2D6*10, CYP2D6*14	*CYP2D6*	Fluvoxamine	Metabolism/PK	Depressive disorder
Level 3	rs35599367	*CYP3A4*	Risperidone	Metabolism/PK	Bipolar disorder, depression, and substance-related disorders
Level 3	rs887829, rs1976391, rs10929302	*UGT1A1*	Risperidone	Toxicity	Autism spectrum disorder
Level 3	rs1045642	*ABCB1*	Risperidone	Toxicity	Schizophrenia
Level 3	rs1128503	*ABCB1*	Risperidone	Efficacy	Autistic disorder
Level 3	CYP2D6*1, CYP2D6*4	*CYP2D6*	Citalopram	Dosage	-
Level 3	rs1065852	*CYP2D6*	Escitalopram	Efficacy	Depressive disorder (major)
Level 3					
Level 3	CYP2D6*1, CYP2D6*4	*CYP2D6*	Sertraline	Dosage	Depression
Level 3	rs2032582	*ABCB1*	Fluoxetine	Efficacy	Depressive disorder

PK, pharmacokinetics.

## 8 Pharmacogenomics interventions in ASD

A very recent PGx study investigated the genetic variants of *CYP1A2* (OMIM ID: 124060), *CYP2C19*, *CYP2D6,* and *SLC6A4* (OMIM ID: 182138) in 42 ASD children who were resistant to pharmacological treatment. The findings of this study revealed that 93% of the ASD children showed improved clinical manifestations after receiving the PGx interventions. Furthermore, 55% of the children in the PGx intervention group achieved stability of clinical symptoms, reducing potential hospital stays and leading to fewer frequent visits to their clinicians. This study suggested that PGx interventions have significant potential to improve the clinical manifestations in severe comorbid ASD children who are resistant to the usual drug treatments (Arranz et al., 2022).

### 8.1 Therapeutic recommendations based on pharmacogenomics testing: updated guidelines

Due to strong genetic associations, pharmacogenomics (PGx)-based dosing guidelines of risperidone clinically indicated for any patients with *CYP2D6* genetic variability have been released by the Dutch Pharmacogenetics Working Group (DPWG) (Beunk et al., 2023). For patients considered *CYP2D6* PMs, the DPWG recommends a dose reduction of risperidone. In contrast, for patients considered *CYP2D6* UMs, the DPWG recommends an alternative antipsychotic drug not primarily metabolized by CYP2D6 or suggests maximizing the dose to achieve the optimum effects (Soria-Chacartegui et al., 2021; Biswas et al., 2022b).

The FDA-approved drug label recommends reducing aripiprazole to 50% of the usual dose for poor *CYP2D6* metabolizers. The DPWG recommends a reduced dose (no more than 10 mg/day or 300 mg/month) for poor *CYP2D6* metabolizers. However, there is no recommendation for intermediate or ultra-rapid metabolizers taking aripiprazole (Dean and Kane, 2012).

## 9 Non-genetic factors

### 9.1 Drug–drug interactions

**FIGURE 5:**
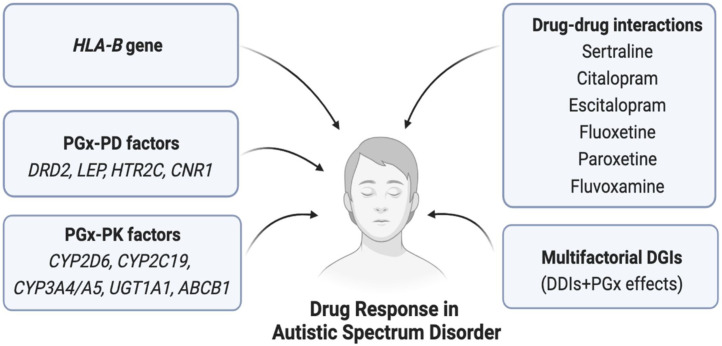
Pharmacogenomics and non-genetic factors affecting drug response in ASD. Here, PGx = Pharmacogenomics, PK = Pharmacokinetics, PD = Pharmacodynamics, HLA = Human leukocyte antigen, DGIs = Drug-gene interactions, DDIs = Drug-drug interactions.

The safety or effectiveness of risperidone in ASD patients may be impacted by clinically significant DDIs, since comorbidities in these patients are likely to be highly prevalent. Risperidone is frequently co-prescribed with antidepressants, anti-epileptics, or other antipsychotics, potentially causing clinically significant DDIs (Puangpetch et al., 2016; Biswas et al., 2022b). Strong CYP2D6 inhibitors (e.g., bupropion) or moderate CYP2D6 inhibitors (e.g., sertraline) may increase the serum concentration of risperidone due to DDIs and potentially cause high blood risperidone-induced adverse effects (Lisbeth et al., 2016). In contrast, CYP2D6 inducer drugs (e.g., rifampin and carbamazepine) may significantly reduce the serum concentration of risperidone if taken concurrently and may cause therapeutic ineffectiveness/failure (Besag and Berry, 2006; Kim et al., 2008; Biswas et al., 2022b). In addition, other mediators affecting the pharmacokinetic properties of risperidone need to be taken into account to avoid potential clinically significant DDIs. For example, the safety or effectiveness of risperidone may be affected if substrate, inhibitor, and inducer drugs of CYP3A4/5 or P-gp are co-prescribed (Kim et al., 2008; Soria-Chacartegui et al., 2021; Biswas et al., 2022b). For aripiprazole, concomitant use of strong CYP2D6/CYP3A4 inhibitors may cause clinically meaningful DDIs, and the prescriber may need to reduce the usual dose of aripiprazole (Dean and Kane, 2012). It is also likely to cause cumulative effects due to the combined DDI and *CYP2D6* genetic effects of risperidone, which may further augment the net clinical effects. Although this multifactorial phenomenon called multifactorial drug–gene interactions (DGIs) is clinically feasible and has been evidenced in cardiovascular drugs, e.g., clopidogrel (Biswas et al., 2021b), such combined effects have not yet been quantified in risperidone therapy. To optimize risperidone therapy, it is, therefore, suggested to consider the risk of both DDIs and pharmacogenomics effects of risperidone in future clinical studies. Some of the clinically significant DDIs of ASD therapies are shown in Table 2. Furthermore, the pharmacogenomic and non-genetic factors affecting drug responses in ASD patients are shown in Figure 5.

**TABLE 2:** Clinically significant DDIs of ASD therapies, as retrieved from the Medscape Drug Interaction Checker (https://reference.medscape.com/drug-interactionchecker).

Main drug	Interacting drug	DDIs and clinical effects	Recommendation
Risperidone	Sertraline	Sertraline may increase the level or effect of risperidone by affecting drug metabolism through the CYP2D6 pathway	Use with caution/monitor
Aripiprazole		Both risperidone and sertraline increase the QTc interval	Use with caution/monitor
Risperidone	Citalopram	Citalopram and risperidone both increase the QTc interval	Avoid or use an alternate drug
		Citalopram will increase the level or effect of risperidone by affecting hepatic enzyme CYP2D6 metabolism	Use with caution/monitor
Risperidone	Escitalopram	Escitalopram increases the toxicity of risperidone by changing the QTc interval	Use with caution/monitor
Aripiprazole			
Risperidone	Fluoxetine	Fluoxetine will increase the level or effect of risperidone by affecting hepatic enzyme CYP2D6 metabolism	Avoid or use an alternative drug
Aripiprazole	Paroxetine	Fluoxetine and risperidone both increase the QTc interval	Use with caution/monitor
Risperidone	Fluvoxamine	Fluvoxamine and risperidone both increase the QTc interval	Use with caution/monitor
Aripiprazole	Citalopram	Aripiprazole and citalopram both increase the QTc interval	Avoid or use an alternative drug

DDIs, drug–drug interactions; ASD, autism spectrum disorder.

## 10 Pharmacogenomics of ASD in Thailand: research and clinical implementation

The prevalence of ASD in Thai children is increasing significantly each year and is potentially increasing the family and social burden (Khaiman et al., 2015; Biswas et al., 2022b). PGx research has progressed considerably in some Asian countries, including Thailand, since many clinically important medications are in routine clinical use in Thailand, where PGx interventions are taken into account (Kloypan et al., 2021; Sukasem et al., 2021; 2023). A large number of clinical studies have already assessed the PGx interference of risperidone in Thai ASD children (Medhasi et al., 2016b; Vanwong et al., 2016b; 2020; Sukasem et al., 2016; 2018; Nuntamool et al., 2017; Srisawasdi et al., 2017; Hongkaew et al., 2018; 2021a; Biswas et al., 2022b). In a prior study, we discovered a significant correlation between the plasma concentration of risperidone and the *CYP2D6* activity score (Vanwong et al., 2016a). These results emphasized the importance of accurately determining a patient’s *CYP2D6* genotype-predicted phenotype in clinical settings for the personalized customization of drug therapy (Vanwong et al., 2016a). In addition to examining *CYP2D6* gene polymorphisms, a previous study aimed at exploring genetic variations in drug-metabolizing enzyme and transporter (DMET) genes associated with steady-state plasma concentrations of risperidone among Thai ASD patients found that *ABCB1* (OMIM ID:171050), *ADH7* (OMIM ID: 600086)*, SLCO1B1* (OMIM ID: 604843)*, SLCO1B3* (OMIM ID: 605495), *SLC7A5* (OMIM ID: 600182), and *UGT2B4* (OMIM ID: 600067) gene polymorphisms were also linked to the plasma concentrations of risperidone. This pharmacogenomic research identified novel genetic variations modulating DMET function that can aid in monitoring risperidone therapy (Medhasi et al., 2016a). In addition, our prior study employed a microarray platform to perform a genetic association analysis of DMET markers with the risperidone-induced prolactin response, evaluated through the hyperprolactinemia and prolactin levels in Thai ASD patients (Hongkaew et al., 2018).

We identified a potential link between *UGT1A1* variants and the prolactin response, which could serve as a foundation for future pharmacogenomic investigations in diverse populations (Hongkaew et al., 2018). In addition to *UGT1A1*, the occurrence of *DRD2 Taq1A* polymorphisms and *DRD2* diplotypes may have a significant effect on the emergence of hyperprolactinemia associated with risperidone use in children and adolescents with a diagnosis of autism spectrum disorder (Sukasem et al., 2016; Hongkaew et al., 2021a). Moreover, the *DRD2* Taq1A polymorphism is linked with a non-stable response to risperidone treatment in patients. This research endorsed the implementation of pharmacogenomics testing to tailor risperidone therapy for individual autistic children and adolescents (Nuntamool et al., 2017). Regarding metabolic adverse effects, a previous study found that gene polymorphisms in leptin and *BDNF* were linked to insulin resistance in Thai children and adolescents with ASD. This implied that leptin and *BDNF* polymorphisms may serve as genetic markers for predicting insulin resistance before commencing treatment in autism spectrum disorder patients receiving risperidone (Sukasem et al., 2018). The overall findings of these studies suggest that PGx screening of some PK/PD genes may be very useful clinically in routine practice to optimize the safety or effectiveness of risperidone in Thai ASD children. Stakeholders and policymakers in Thailand should now focus on the preparation of national PGx guidelines based on the robust evidence from these studies, especially regarding risperidone for Thai ASD children as part of precision medicine (Biswas et al., 2022b).

## 11 Challenges in pharmacogenomic implementation

### 11.1 *CYP2D6* discrepancy

The *CYP2D6* allele activity score (AS) varies greatly, and this discrepancy may affect the designation of predicted phenotypes, as discussed extensively in our previous review (Biswas et al., 2022b).The AS of different *CYP2D6* alleles is shown in Supplementary Table S1. The assignment of predicted phenotypes based on the AS of *CYP2D6* has been discussed previously (Biswas et al., 2022b), and the predicted phenotypes based on the combined *CYP2D6* allele AS are shown in Supplementary Table S2.

Novel alleles (i.e., CYP2D6*142, *143, and *144) and a novel sub-allele (CYP2D6*10.005) were discovered in the Thai population and have already been recognized by the PharmVar (Hongkaew et al., 2021c), but the ASs of these novel alleles have not yet been assigned. Since the AS may vary, which may affect the assignment of predicted phenotypes accordingly, it is suggested to assess the function of *CYP2D6* genetic variants by measuring the protein expression level for further validation of the predicted phenotypes (Biswas et al., 2022b).

### 11.2 Polygenic risk score

When multiple genetic variants are involved in determining the clinical response of a drug, the polygenic risk score (PRS) may be a good predictor to assess the safety or effectiveness of that particular drug rather than just considering the effects of each genetic variant separately. The polygenic pharmacogenomics response model might be an integral part of precision medicine development, especially when more than one potential genetic variant will tailor the safety or effectiveness of medications (Lewis et al., 2019; Biswas, 2021; Ikeda et al., 2021). Since multiple PK/PD genetic variants may modify the clinical response of risperidone/aripiprazole, the PRS approach would be suitable for these drugs and should be considered in future clinical studies.

## 12 Future perspectives and opportunities

### 12.1 Pharmacogenomics guidelines

The DPWG has already published PGx-based dosing guidelines for atypical antipsychotics, i.e., risperidone and aripiprazole, not just for ASD, but for all other clinical conditions where these drugs are clinically indicated. However, other international PGx working groups, such as CPIC and CPNDS, have not yet published any guidelines for either risperidone or aripiprazole. In the near future, it is expected that other PGx working groups will publish guidelines to facilitate the translation of risperidone/aripiprazole PGx into routine clinical practice (Biswas et al., 2022b). Our group assigned a *CYP2D6* score of ‘1’ as NM in risperidone due to the comparative blood concentration levels of risperidone. However, a *CYP2D6* score of ‘I’ was assigned as IM by the recent CPIC guidelines for selective serotonin reuptake inhibitors (SSRIs) instead of NM (Hongkaew et al., 2021b; Bousman et al., 2023). The controversy regarding *CYP2D6* scoring systems and predicted phenotypes for risperidone metabolism that has arisen between “the consortium (CPIC + DPWG)” and “the PPM-pharmacogenomics of autism spectrum disorders of the Thailand Project” is shown in Table 3. The government of Thailand should consider the *CYP2D6* scoring system for risperidone as suggested by the PPM Laboratory, since it may be highly applicable for Thai patients and may expedite the formation of prescribing guidelines, which may further help to improve the safety or effectiveness of risperidone in ASD.

**TABLE 3:** Controversy regarding the CYP2D6 activity score range and predicted phenotype for the risperidone metabolism rate between “the consortium (CPIC + DPWG)” and “the PPM-pharmacogenomics of autism spectrum disorders of Thailand Project.”

*CYP2D6* predicted phenotype based on the combined score	*CYP2D6* activity score range (CPIC + DPWG)	*CYP2D6* activity score range for risperidone in Thai: ASD
Ultra-rapid metabolizer (UM)	>2.25	>2.0
Normal metabolizer (NM)	1.25, 1.5, 2.0, 2.25	**1.0**, 1.25, 1.5, 2.0
Intermediate metabolizer (IM)	0.25, 0.5, 0.75, **1.0**	0.25, 0.5, 0.75
Poor metabolizer (PM)	0	0

CPIC, clinical pharmacogenetics implementation consortium; SSRIs, selective serotonin reuptake inhibitors; PPM, pharmacogenomics and precision medicine, Ramathibodi Hospital, Mahidol University.

### 12.2 Clinical implementation

Many factors, including infrastructure and robust evidence, are involved in the successful implementation of PGx in routine clinical practice. Precision medicine for ASD may be achieved through ensuring PGx screening for at least *CYP2D6* genetic variants in routine clinical practice (Biswas et al., 2022b).

### 12.3 Reimbursement

Often, reimbursement for genetic testing hinders the clinical implementation of PGx. Reimbursement coverage should be applied for ASD medications, or at least for risperidone, with wider clinical adoption (Biswas et al., 2022b).

### 12.4 Undetermined and rare SNPs

Novel genes and SNPs should be considered. WGS can help with their discovery, such as our finding of novel SNPs in the discrepancy between the risperidone level and *CYP2D6* genotyping, leading to the determination of novel *142, *143, and *144 in an ASD study (Hongkaew et al., 2021c).

### 12.5 Healthcare provider awareness and knowledge

Healthcare professionals must be aware of the PGx associations of ASD medications and, obviously, must have adequate knowledge about the PGx interference of ASD medications. Along with pharmacists, doctors are the main driving force behind the implementation of newly evolving PGx approaches in real clinical practice (Albassam et al., 2018; Edris et al., 2021; Biswas et al., 2022b). Since many of these healthcare professionals do not have sufficient knowledge or confidence to implement precision medicine in clinical settings, education and trainings are obvious to make them professionally competent. A recent study concluded that an adaptable and flexible training module is needed for targeted healthcare professionals for the successful implementation of precision medicine in routine clinical practice (Mitchell et al., 2022).

## 13 Conclusion

Although there is no selective pharmacotherapy for ASD, the FDA has recommended risperidone/aripiprazole to treat associated symptoms of ASD, such as agitation/irritability. Strong associations of some pharmacokinetic/pharmacodynamic gene variants, e.g., *CYP2D6* and *DRD2*, with risperidone-induced hyperprolactinemia have been found in children with ASD, but such genetic associations have not been found directly for aripiprazole in ASD. In addition to PGx factors, DDIs and possibly the cumulative effects of DDIs and PGx, called multifactorial DGIs, may regulate the safety or effectiveness of risperidone/aripiprazole, which should be assessed in future clinical studies in children with ASD. Reimbursement, knowledge, and education of healthcare professionals are the key obstacles preventing the successful implementation of ASD pharmacogenomics into routine clinical practice. The preparation of national and international PGx-based dosing guidelines for risperidone/aripiprazole based on robust evidence may advance the precision medicine of ASD.
