# Supplementary material for: Pharmacogenomics and non-genetic factors affecting drug response in autism spectrum disorder in Thai and other populations: current evidence and future implications
Source: Front Pharmacol. 2024 Feb 5;14:1285967. doi: 10.3389/fphar.2023.1285967 (PMC10875059; doi:10.3389/fphar.2023.1285967)
Supplement: Supplementary file 1 [file Table1.docx]

**Supplementary Table S1.** The score assignment for *CYP2D6* alleles.

| ***CYP2D6* alleles** | **Score assigned to allele** |
| --- | --- |
|  |  |
| **3, *4, *5, *6, *7, *8, *11, *12, *13, *18, *19, *20, *21,*31, *36, *38, *40, *42, *44, *47,*51, *56, *57, *60, *62, *68, *69, *81, *92, *96, *99, *100, *101, *114, *120, *124, *129, *143, *144, *156, *161* and duplication of no functional alleles *(*e.g.,**4xN, *5xN)* | 0 |
| **10* | 0.25 |
| **9, *14, *15, *17, *29, *32, *41, *45, *46, *49, *50, *52, *54, *55, *59, *109, *119, *132* | 0.5 |
| **1, *2, *27, *33, *34, *35, *35, *39, *43, *45, *46, *48, *53,* | 1 |
| Duplication of normal functional alleles *(*e.g., **1xN, *2xN)* | 2 |

*Source: https://www.pharmvar.org/gene/CYP2D6*
