# Supplementary material for: Pharmacogenomics and non-genetic factors affecting drug response in autism spectrum disorder in Thai and other populations: current evidence and future implications
Source: Front Pharmacol. 2024 Feb 5;14:1285967. doi: 10.3389/fphar.2023.1285967 (PMC10875059; doi:10.3389/fphar.2023.1285967)
Supplement: Supplementary file 2 [file Table2.docx]

**Supplementary Table S2**. Comparisons of *CYP2D6* activity scoring system and associated predicted phenotypes.

| **Combined *CYP2D6* allele activity score range** | | | ***CYP2D6* predicted phenotype based on combined score** | **Examples**  **Of *CYP2D6* diplotypes** |
| --- | --- | --- | --- | --- |
| **DPWG activity score range** | **CPIC activity score range** | **Revised and updated activity score range** |  |  |
| >2.5 | >2 | >2.25 | Ultrarapid metabolizer (UM) | **1/*1xN, *1/*2xN* |
| 1.5–2.5 | 1–2 | 1.25–2.25 | Normal metabolizer (NM) | **1/*1, *2/*2, *2/*10* |
| 0.5–1 | 0.5 | 0.25–1 | Intermediate metabolizer (IM) | **10/*10, *36+10/*14, *1/*5, *2/*4* |
| 0 | 0 | 0 | Poor metabolizer (PM) | **5/*5, *4/*4xN* |

Here, DPWG=Dutch Pharmacogenetics Working Group; CPIC=Clinical Pharmacogenetics Implementation Consortium. This table has been updated and adapted from Nofziger C *et al.,* 2020 and Hongkaew Y *et al.,* 2021 (Hongkaew, Gaedigk, Wilffert, Ngamsamut, et al., 2021; Nofziger et al., 2020).
